# Novel HCV Genotype 4d Infectious Systems and Assessment of Direct-Acting Antivirals and Antibody Neutralization

**DOI:** 10.3390/v14112527

**Published:** 2022-11-15

**Authors:** Long V. Pham, Rodrigo Velázquez-Moctezuma, Ulrik Fahnøe, Laura Collignon, Priyanka Bajpai, Christina Sølund, Nina Weis, Kenn Holmbeck, Jannick Prentoe, Jens Bukh

**Affiliations:** 1 Copenhagen Hepatitis C Program (CO-HEP), Department of Infectious Diseases, Copenhagen University Hospital, Hvidovre and Department of Immunology and Microbiology, Faculty of Health and Medical Sciences, University of Copenhagen, DK-2200 Copenhagen, Denmark; 2 Department of Infectious Diseases, Copenhagen University Hospital, DK-2650 Hvidovre, Denmark; 3 Department of Clinical Medicine, Faculty of Health and Medical Sciences, University of Copenhagen, DK-2200 Copenhagen, Denmark

**Keywords:** hepatitis C virus, genotype 4d, infectious culture system, direct-acting antivirals, neutralizing antibodies, human-liver chimeric mice, Huh7.5 cells

## Abstract

Hepatitis C virus (HCV) genotype 4 is highly prevalent in the Middle East and parts of Africa. Subtype 4d has recently spread among high-risk groups in Europe. However, 4d infectious culture systems are not available, hampering studies of drugs, as well as neutralizing antibodies relevant for HCV vaccine development. We determined the consensus 4d sequence from a chronic hepatitis C patient by next-generation sequencing, generated a full-length clone thereof (pDH13), and demonstrated that pDH13 RNA-transcripts were viable in the human-liver chimeric mouse model, but not in Huh7.5 cells. However, a JFH1-based DH13 Core-NS5A 4d clone encoding A1671S, T1785V, and D2411G was viable in Huh7.5 cells, with efficient growth after inclusion of 10 additional substitutions [4d(C5A)-13m]. The efficacies of NS3/4A protease- and NS5A- inhibitors against genotypes 4a and 4d were similar, except for ledipasvir, which is less potent against 4d. Compared to 4a, the 4d(C5A)-13m virus was more sensitive to neutralizing monoclonal antibodies AR3A and AR5A, as well as 4a and 4d patient plasma antibodies. In conclusion, we developed the first genotype 4d infectious culture system enabling DAA efficacy testing and antibody neutralization assessment critical to optimization of DAA treatments in the clinic and for vaccine design to combat the HCV epidemic.

## 1. Introduction

Hepatitis C virus (HCV) infection is a major cause of chronic hepatitis, which eventually can lead to liver cirrhosis and hepatocellular carcinoma (HCC). The virus contains a positive-stranded RNA genome that consists of an open-reading frame (ORF) flanked by 5′-and 3′-untranslated regions (UTRs), critical for viral replication [1]. The ORF encodes a polyprotein that is further processed into 10 mature proteins Core, E1, E2, p7, NS2, NS3, NS4A, NS4B, NS5A, and NS5B [1]. The HCV genome is highly heterogenous and classified into eight different genotypes and >90 subtypes [2,3,4]. Globally, genotype 4 accounts for ~8% of HCV infections [2], with subtypes 4a and 4d being most prevalent [5]. The HCV subtype 4d, originally identified in a patient from Denmark, has recently emerged in Europe [2,6]. This subtype is frequently transmitted among intravenous drug users and in association with men having sex with men [2,5]. Although both subtypes 4a and 4d are common among genotype 4 isolates, the clinical investigation has focused mainly on 4a.

Combinations of direct-acting antivirals (DAAs), targeting NS5A and the NS3/4A protease (PI), or NS5A and the NS5B polymerase [7], have revolutionized the treatment of HCV infection with an impressive cure rate resulting in a sustained virological response (SVR) of >95%, defined as negative HCV RNA 12 weeks after end of treatment [8]. Nevertheless, one of the remaining challenges for DAA treatment is viral resistance due to the naturally occurring polymorphism and development of resistance-associated substitutions (RAS) [8]. Although treatment failure rates are low, the number of patients who fail DAA treatment will increase significantly when huge numbers are treated worldwide. For genotype 4, SVR could be achieved by ≥90% [9]. However, treatment failures have been widely reported for genotype 4d [10], due to prevalence in people with high-risk behaviors who are prone to re-infection. This could increase the emergence and spread of RASs in genotype 4d infections compared to that in genotype 4a [11,12]. Thus, careful monitoring of DAA failures would be necessary to prevent the emergence and spread of HCV resistance variants, especially in high-risk populations. In this context, HCV culture models provide valuable tools for understanding the emergence of RASs, as well as viral escape and barrier to resistance.

Although DAAs are highly effective therapies, these might not necessarily prevent re-infections, which could hamper the elimination of HCV. In addition, protective immunity would be an important factor for protection against new infections. Worldwide, about 2 million acute infections have been reported annually, and a vaccine would therefore be an important tool for achieving the WHO goal of eliminating HCV as a public health threat. One of the key objectives after vaccination is to induce neutralizing antibodies (NAbs) to gain protective immunity [13]. Further, NAbs play an important role in clearance of HCV in the acute phase [14], and studies from animal models have shown that NAbs can indeed provide protection against HCV infection [15]. However, the efficacy of the immune response induced by NAbs could be influenced by viral heterogeneity, and even NAbs with broad activity primarily protect against challenge with the homologous HCV virus [16]. In addition, the high mutational rates during HCV replication could enable the virus to evolve to escape from NAbs [17], as mutations accumulated in the envelope E1 and E2 proteins have been shown to render the virus resistant to NAbs [18,19,20]. Thus, it is highly relevant and important to gain more knowledge about induction of NAbs in real-life infection and vaccine development, which could help to better define the role of NAbs. In this context, experimental systems for studies of NAbs are of great importance. Two in vitro models to study HCV neutralization include pseudo-particles expressing HCV E1 and E2 proteins (HCVpp) and HCV infectious cell culture (HCVcc) systems. The HCVpp system permits studies of viral entry, investigation of entry receptors, and evaluation of neutralization capacity [13,21,22]. Nevertheless, the HCVpp system does not enable studies identifying mutation-induced viral escape or addressing other steps of the viral life cycle beyond viral entry. These aspects are better explored by use of HCVcc, which recapitulates the full viral life cycle. The HCVcc systems are likewise valuable for studying efficacy of NAbs against viral infection in vitro. In addition, the HCVcc models are ideally suited for investigating viral escape from and barrier of resistance to NAbs [23].

Given the advantages of infectious HCV cell culture systems in studies of antivirals and for vaccine development, there have been tremendous efforts to develop such systems. However, it remains a great challenge to develop infectious culture systems for all HCV subtypes, since patient isolates are not viable in culture. Further, even though HCV clones generated from consensus sequences of patient isolates could be infectious in vivo, such clones are rarely viable in cell culture, except for the JFH1 strain (genotype 2a), which can propagate in culture [24,25]. Taking advantage of this feature of JFH1, various chimeric recombinants have been developed. The JFH1-based chimeras with Core-NS2 from other genotypes were generated, providing useful tools for studying viral entry and neutralization of different genotypes [23,24,26,27,28,29]. Although these chimeras are replication competent in cell culture, adaptive mutations are required for efficient propagation, except for Core-NS2 recombinants of genotypes 2a, 2b, and 2c [23,24,30]. The most advanced JFH1-based chimeras are recombinants expressing genotype-specific sequences of Core-NS5A (C5A) or 5′UTR-NS5A (55A), which are highly valuable for studies of DAA regimens such as PIs and NS5A inhibitors individually and in combinations [24,31,32,33]. Generally, these chimeras are non-viable or highly attenuated in cell culture, thus also requiring acquisition of adaptive mutations to robustly replicate and produce infectious particles [24]. Importantly, mutations identified in 55A and C5A recombinant viruses are highly useful in the process of developing the JFH1 independent full-length recombinant virus, which are unique models to study different aspects of viral life cycle independently of JFH1 [24]. However, the development of such systems remains an extremely difficult task, and a universal strategy to achieve this ultimate goal has not been described. The process of virus adaptation that relies on cell culture acquired mutations has remained state of the art for culturing full-length HCV efficiently. Although the acquired mutations identified from adapted JFH1-based chimera, especially C5A and 55A recombinants, have been instrumental for developing efficient full-length infectious systems, such mutations would not typically be enough to confer viability of full-length recombinants in cell culture because most adapted full-length recombinant viruses require additional mutations in NS5B [24]. The genotype - specific NS5B mutations are crucial for viability of full-length viruses, but identification of such mutations is challenging and time-consuming. So far, there are only few efficient full-length infectious systems, developed for genotypes 1a, 1b, 2a, 2b, 2c, 3a, 4a, and 6a [24,32,33,34,35,36,37,38,39,40,41,42,43]. However, the infectious cell culture systems for HCV genotype 4d have not been developed.

Here, we aimed at filling this void by developing HCV genotype 4d infectious cell culture systems and using these new systems to test efficacy of DAAs and neutralization capacity of broadly reactive human monoclonal NAbs and NAbs in plasma from HCV genotype 4a and 4d infected patients. In addition, using the novel 4d infectious systems, we investigated the neutralization capacity of patient plasma taken during the infection and after successful DAA treatments in a patient with two successive HCV genotype 4d infections.

## 2. Materials and Methods

### 2.1. Construction of HCV Genotype 4d Clone

The consensus ORF sequence of a HCV genotype 4d strain, named DH13, was obtained from a chronic hepatitis C patient plasma sample, taken before the patient underwent DAA treatment. This patient, co-infected with HIV, was recruited into The Danish Database for Hepatitis B and C (DANHEP). The whole HCV ORF was amplified by reverse-transcription PCR (RT-PCR) using the same primers that are used for amplification of genotype 4a ED43 [34]. The PCR product was subjected to next-generation sequencing (NGS) analysis to determine the consensus sequence. A consensus ORF clone was generated from clones obtained by TOPO-XL cloning (ThermoFisher, Waltham, MA, USA) by assembling fragments using InFusion cloning (Takara, Kusatsu, Shiga, Japan). The ORF was then cloned into a pED43 vector containing genotype 4a ED43 5′- and 3′-UTRs [34]. The 4d Core-NS5A (C5A) clone was constructed by InFusion assembly of the 4d DH13 consensus Core-NS5A sequence into the J6/JFH1 plasmid containing JFH1 NS5B and both UTRs [26]. The JFH1-based genotype 4a ED43 Core-NS2 recombinant, which contains ED43 Core-NS2, was described previously [44]. Mutations were introduced by QuikChange site-directed mutagenesis (Agilent, Santa Clara, CA, USA) or megaPCR approach [45]. All HCV sequences of the final plasmids were sequence confirmed (Macrogen, Seoul, Republic of Korea). The nucleotide (nt) and amino acid (aa) numbers refer to the full-length 4d DH13 clone sequence.

### 2.2. Determination of 5′- and 3′UTR of Genotype 4d

Amplification of the 5′UTR of genotype 4d DH13, obtained from patient plasma, was performed as described previously [46] with some modifications. Synthesis of cDNA was carried out at 50 °C for 1 h using Maxima H Minus Reverse Transcriptase (ThermoFisher, Waltham, MA, USA) and a 4d core-specific reverse primer 4d-1320-R (Table 1). The cDNA was purified, tailed with dA, and the first round PCR was performed using Oligo dT-anchor primer (5′/3′ RACE Kit, second generation; Roche, Basel, Switzerland) and a 4d core-specific reverse primer 4d-540-R (Table 1). The first round PCR product was used as a template for the second round PCR using anchor primer (5′/3′ RACE Kit, second generation; Roche, Basel, Switzerland) and a 4d core-specific reverse primer 1a1b4aR352 [46]. The amplified product was gel extracted and Sanger sequenced.

To determine the sequence of the variable region and poly (U/C) tract of genotype 4d DH13 3′UTR, extracted viral RNA from patient plasma was denatured at 70 °C for 5 min followed by cDNA synthesis at 50 °C for 1 h using Maxima H Minus Reverse Transcriptase (ThermoFisher, Waltham, MA, USA) and a genotype 4a X-region specific reverse primer 4drX-9584-RT (Table 1). PCR amplification of cDNA was performed using Q5 Hot Start High-Fidelity 2X Master Mix (New England Biolabs, Ipswich, MA, USA) and primers 4dr-8530-F and 4drX-9562-R (Table 1). PCR conditions were 98 °C for 30 s, followed by 40 cycles of 98 °C for 10 s, 66 °C for 10 s, and 72 °C for 1 min, with a final extension at 72 °C for 5 min. The amplified product was gel extracted and cloned into pCR-XL-2-TOPO vector (ThermoFisher, Waltham, MA, USA), according to manufacturer’s recommendations. Sequence of the insert was determined by Sanger sequencing.

To determine the sequence of X-region of genotype 4d DH13 3′UTR, the extracted viral RNA was tailed with homopolymers of ATP at 37 °C for 10 min using Yeast Poly(A) Polymerase (Thermo Scientific, Waltham, MA, USA), followed by synthesis of cDNA using Maxima H Minus Reverse Transcriptase (ThermoFisher, Waltham, MA, USA), and primer TS-O-00178 (Table 1) [47]. Briefly, the A-tailed RNA was incubated at 75 °C for 3 min and ramped down to 50 °C before adding Maxima H Minus Reverse Transcriptase. Synthesis of cDNA was performed in a continuous gradient from 50 °C to 55 °C for 60 min, followed by PCR amplification using Q5 Hot Start High-Fidelity 2X Master Mix (New England Biolabs, Ipswich, MA, US), and primer 3UTR-9476-F1 (Table 1) and reverse primer AUAP (Table 1; 5′ RACE System; ThermoFisher, Waltham, MA, USA). PCR conditions were 98 °C for 30 s, followed by 40 cycles of 98 °C for 10 s, 53 °C for 10 s, and 72 °C for 1 min, with a final extension at 72 °C for 5 min. The amplified product was cloned into pCR™4Blunt-TOPO™ vector (ThermoFisher, Waltham, MA, USA), according to manufacturer’s instructions. The clones were Sanger sequenced.

### 2.3. Analysis of Virus Recovered from Cell Culture

The human hepatoma cell line Huh7.5 was cultured in Dulbecco’s Modified Eagle’s Medium (DMEM) supplemented with 10% fetal bovine serum (Sigma-Aldrich, St Louis, MO, USA) and 100 units/mL of penicillin-streptomycin (ThermoFisher, Waltham, MA, USA). All cultures were maintained at 37 °C and 5% CO_2_. To analyze viral viability of recombinants, in vitro RNA transcripts were transfected into Huh7.5 cells using lipofectamine 2000 (ThermoFisher, Waltham, MA, USA), and the presence of HCV antigen positive cells were determined by immunostaining with anti-HCV NS5A antibody [26] and anti-HCV core antibody (Enzo life sciences, Farmingdale, NY, USA). The transfected cultures were sub-cultured every 2–3 days, as described [34]. When virus spread (≥90% HCV positive cells), the supernatants were collected and stored at −80 °C. Viral passages were performed, and infectivity titers were determined by focus-forming unit (FFU) assays and shown as log_10_FFU/mL, as described previously [34]. To determine the sequences of recovered viruses, supernatants were used for RNA extraction and RT-PCR, subsequently. The whole ORFs were obtained and analyzed by NGS, as described above [48].

### 2.4. Treatment with Direct-Acting Antivirals (DAAs)

All dose-response assays with NS3A/4A protease- and NS5A- inhibitors were carried out as described previously [34,49]. Briefly, Huh7.5 cells were seeded on 96-well plates and infected with supernatant viral stocks for 24 h. Subsequently, serial dilutions of indicated inhibitors were added to the plates and incubated for an additional 48 h. The plates were fixed and immunostained. The number of HCV positive cells were counted automatically using the ImmunoSpot series 5 UV analyzer. A sigmoidal concentration-response curve was fitted using GraphPad Prism 9 and the half-maximal effective concentration (EC_50_) values were calculated.

### 2.5. HCV Neutralization Assay

Neutralization was carried out as described [50]. Briefly, a final readout of 50 to 200 FFU of HCV were incubated either with human monoclonal antibodies AR3A and AR5A, or plasma obtained from HCV genotype 4a or 4d chronically infected patients, in a single dilution 1/50 or dilution series, for 1 h at 37 °C. Following this pre-incubation, antibody–virus mixes or virus only were added to 6 × 10^3^ Huh7.5 cells plated the day before in poly-D-lysine-coated 96-well plates. Following 4 h infection at 37 °C, the cells were washed and incubated in fresh medium for a total infection time of 48 h. Cells were then fixed and stained with anti-HCV NS5A antibody [26]. The data were normalized to 6 replicates of virus only for single-dose neutralization or 8 replicates of virus only for dilution series neutralization. Dose-response neutralization experiments were further analyzed using four-parameter curve fitting in GraphPad Prism 9.

### 2.6. Analysis of HCV Viability in Human-Liver Chimeric Mice

Male and female Alb-uPA mice were maintained in a CB-17/Icr-Prkdcscid/scid/Rj (SCID) background with ad libitum access to food (SAFE D03, SAFE Complete Care Competence, Rosenheim, Germany) and water. The animals were housed by gender (except for breeders) in Innovive IVC caging containing wood chip bedding, shelters, nesting material, and biting sticks on a 12-h light dark cycle. All experimentation was conducted during light cycle adhering to 3R principles and with procedures consistent with affirmative response to the ARRIVE 10 questionnaire. Human-liver chimeric mice were generated as previously described [51,52]. Briefly, homozygous uPA-SCID mice were transplanted with cryopreserved primary human hepatocytes (donor HUM191501, Lonza, Basel, Switzerland) via intrasplenic injection under isoflurane anesthesia. Six weeks after transplantation, successful engraftment was assessed by human albumin concentration in mouse plasma, determined by ELISA (Bethyl Laboratories, Montgomery, TX, USA).

For intrahepatic inoculation of animals with full-length HCV RNA transcripts, the plasmid containing the 4d DH13 full-length clone (pDH13) was prepared using EndoFree Plasmid Maxi Kit (Qiagen, Hilden, Germany) and sequence confirmed. One mouse was inoculated under isoflurane anesthesia with 10 μg of PBS diluted in vitro RNA transcript of pDH13. Blood was sampled weekly by vein puncture under anesthesia according to guidelines for blood sampling, processed for serum, and stored at −80 °C until use.

Total RNA was extracted from mouse serum using TRIzol LS/chloroform extraction, purified with the RNA Clean & Concentrator kit (Zymo Reseach, Irvine, CA, USA) and eluted in 25 µL nuclease-free water. To quantify HCV RNA, a one-step real-time RT-qPCR reaction was performed using the LightCyler 480 (Roche, Basel, Switzerland), the TaqMan^®^Fast Virus 1-Step Master Mix (ThermoFisher, Waltham, MA, USA), and a primers/probe set listed in Table 1 [53]. A standard curve was generated by 10-fold dilutions of in vitro transcribed RNA of the H77/JFH1 recombinant virus after two successive steps with DNase treatments and purification with RNeasy kit (Qiagen, Hilden, Germany) [44].

The sequences of HCV ORF and UTRs were determined as described in Section 2.1 and Section 2.2.

## 3. Results

### 3.1. In Vivo and In Vitro Analysis of HCV Genotype 4d Full-Length Clone

We initially determined the full-length ORF consensus sequence of HCV genotype 4d (strain name: DH13) from a hepatitis C patient co-infected with HIV (see Materials and Methods) (Genbank accession number OP555741). The genome translates into a polyprotein of 3006 aa. Compared to 11 genotype 4d sequences listed in the Los Alamos database (retrieved on 31 October 2022), the DH13 4d ORF shows 92–97% and 94–97% identities at nt and aa levels, respectively [54]. We also determined the UTR sequences of DH13 (OP555741). For the 5′UTR, there are 2 complete and 3 partial 4d sequences in Los Alamos and euHCV databases, which is identical with the DH13 4d 5′UTR sequence [54,55]. For the 3′UTR, there is no complete 4d sequence available in Los Alamos and euHCV databases [54,55].

**Figure 1:**
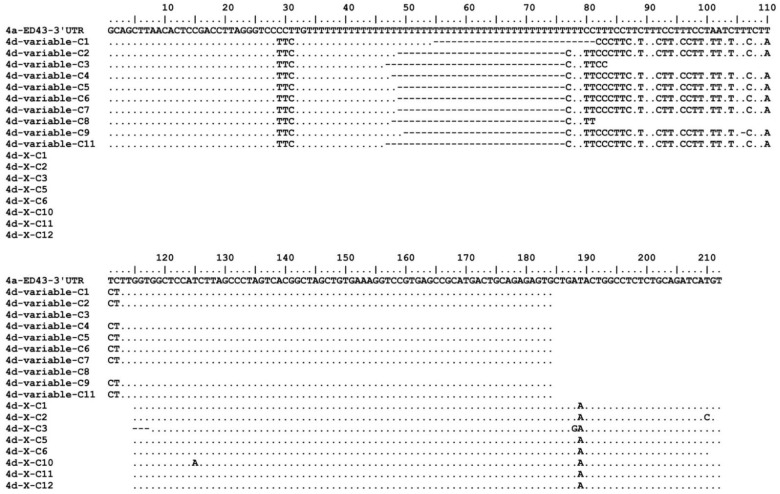
Analysis of DH13 (HCV genotype 4d) 3′ UTR. The PCR amplicons of DH13 3′-variable, poly-UC, and X-region were obtained by RT-PCR from a hepatitis C patient infected with genotype 4d (see Materials and Methods). The amplicons were cloned into TOPO vectors and were subsequently Sanger sequenced. A total of 18 clones (10 clones for variable-region/poly-UC and 8 clones for X-region) covering the entire 3′UTR sequence were analyzed and aligned with the ED43 (4a) 3′UTR [34,56]. The ORF stop codon was excluded from this alignment. Dots, identical with ED43 sequence; dashes, shorter poly-UC compared with ED43 sequence.

Compared with the 4a ED43 prototype sequence [56], the 4d ORF shows 80% and 88% identities at nt and aa levels, respectively. For the 5′UTR, we found one change of T203A compared with ED43 [46]. In the 3′UTR, the variable region was conserved between ED43 (4a) and DH13 (4d) (Figure 1). The X-region was also highly conserved with only one change when comparing with the genotype 4a ED43 clone (the T189A change in Figure 1) [56]. However, this residue of ED43 3′X, determined by Kolykhalov et al., has nucleotide A [57]. In contrast, the poly(U(T)/C) tract of DH13 (4d) is shorter and contained several differences compared with ED43 (4a) (Figure 1) [56].

We decided to generate a genotype 4d full-length clone containing genotype 4a ED43 5′- and 3′-UTRs (Genbank accession number OP555742), since the ED43 UTRs have been shown to confer viability of genotype 4a both in vivo and in vitro [34,56]. This genotype 4d full-length clone (pDH13) was in vitro transcribed and RNA-transcripts were transfected intrahepatically into a uPA-SCID mouse that was transplanted with cryopreserved primary human hepatocytes. We followed the animal for 4 weeks after transfection, and HCV RNA titers in serum were 6.1-, 7.7-, 8.1-, and 7.9 log_10_ genome equivalents per ml (GE/mL) at week 1, 2, 3, and 4, respectively. NGS analysis of the ORF sequence of virus recovered from the animal at week 4 showed that no additional coding mutations emerged with a frequency ≥5% (data not shown). We also analyzed the 5′- and 3′-UTR sequences from recovered viruses and confirmed that the sequences were maintained. These results indicated that the genotype 4d full-length DH13 clone was fully viable in vivo.

Using Huh7.5 cells, we next tested whether pDH13 was viable in vitro. However, after transfection of Huh7.5 cells with RNA transcripts, we could not detect any HCV antigen-positive cells. In two independent transfections, the full-length 4d recombinant remained non-viable during 2 weeks of follow-up. Therefore, we attempted to identify adaptive mutations permitting culture of pDH13. We have reported that recombinants with genotype-specific C5A, JFH1-NS5B, and JFH1-UTRs could be adapted, and the adaptive mutations identified in C5A recombinant viruses could also be valuable for subsequent development of full-length recombinant virus [24,32,33,34]. Thus, we next generated a DH13-C5A recombinant for this purpose.

### 3.2. Development of JFH1-Based Genotype 4d Core-NS5A Infectious System

**Figure 2:**
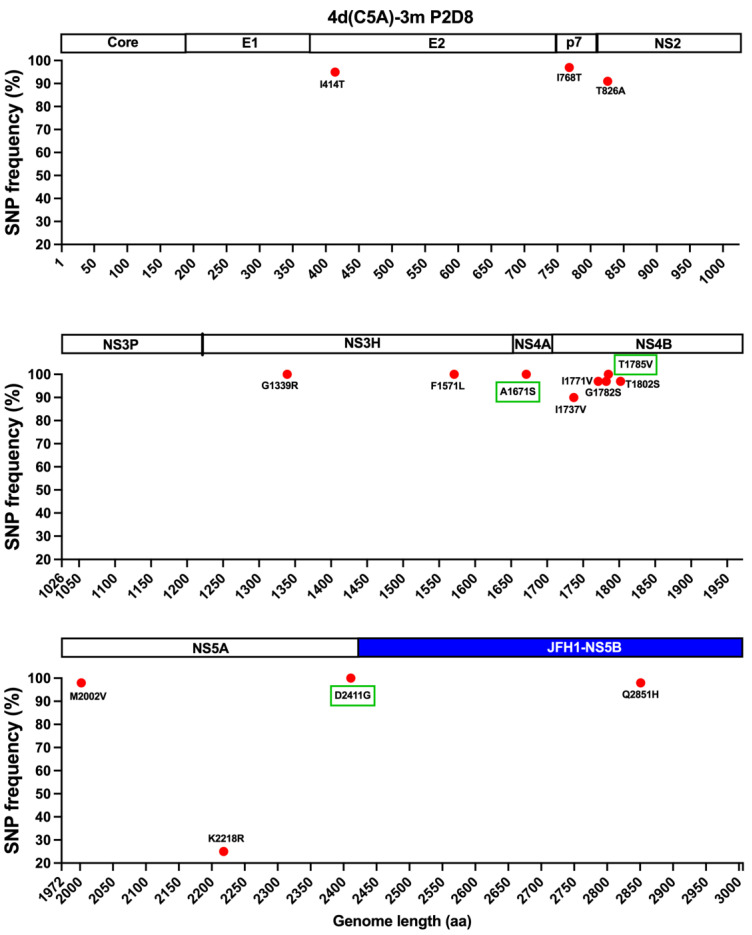
Next generation sequencing (NGS) analysis of the recovered JFH1-based 4d Core-NS5A (C5A) virus. The 4d(C5A)-3m recombinant virus, which contains JFH1 NS5B and UTRs, recovered from the 2nd passage (P2), was subjected to NGS analysis (full coding sequence is split into three equal parts in the figure). The SNP frequencies (%) for mutations that developed ≥20% are shown with corresponding HCV proteins (Core: 1–191; E1: 192–383; E2: 384–745; p7: 746–808; NS2: 809–1025; NS3: 1026–1656; NS4A: 1657–1710; NS4B: 1711–1971; NS5A: 1972–2415; NS5B: 2416–3006). The green boxes indicate mutations that were originally introduced in the 4d(C5A)-3m recombinant. P2D8, day 8 in the 2nd passage. aa, amino acid. The region of JFH1-NS5B is indicated with blue bar.

The JFH1-based DH13 Core-NS5A recombinant, henceforth designated 4d(C5A), did not yield any HCV antigen-positive cells during 2 weeks of follow-up after transfection of Huh7.5 cells with in vitro RNA transcripts. Thus, mutations are required for viability of the JFH1-based 4d(C5A) recombinant. We therefore introduced mutations A1671S(NS4A) and T1785V(NS4B) into 4d(C5A) (designated 4d(C5A)-2m). These mutations enabled viability in a genotype 4a(C5A) ED43 recombinant [34]. In addition, we introduced a previously identified mutation by cell culture adaptation, D2411G(NS5A) into 4d(C5A)-2m, generating 4d(C5A)-3m [24]. After transfection of Huh7.5 cells, both 4d(C5A)-2m and 4d(C5A)-3m recombinants yielded HCV antigen-positive cells. The 4d(C5A)-2m culture was stopped after 2 months of follow-up since we did not observe viral spread. The 4d(C5A)-3m culture was continued, and the virus spread at day 80. The virus supernatant of this recombinant could infect naïve Huh7.5 cells. In the 2nd passage, the recombinant virus spread (≥90% HCV positive cells) at day 8 and produced infectivity titer of ~4.3 log_10_ FFU/mL. NGS analysis of recovered virus revealed that 12 mutations emerged with frequency ≥20% in the viral population, while the 3 introduced mutations were maintained at ~100% (Figure 2).

**Figure 3:**
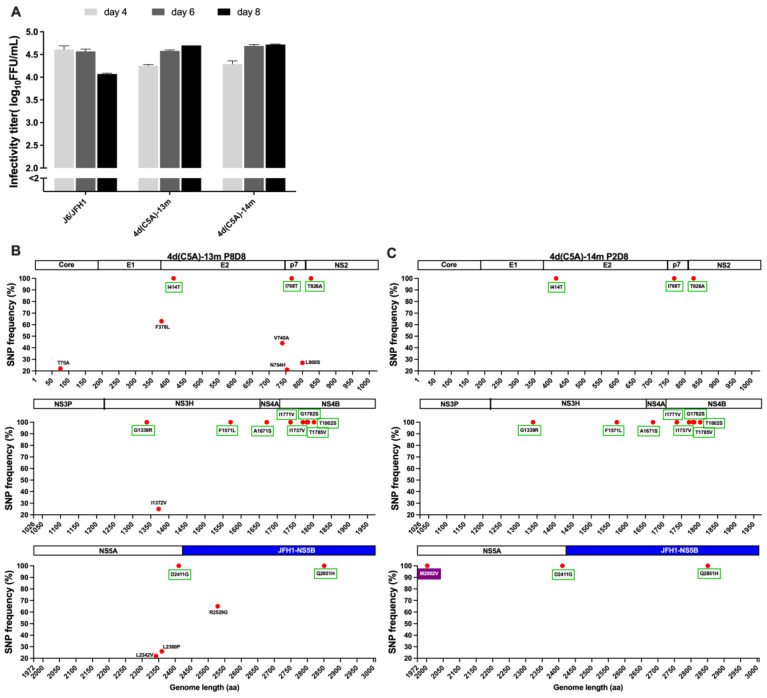
Viability and NGS analysis of 4d(C5A) recombinant viruses. (**A**): Infectivity titers in supernatants for selected time points of indicated viruses were determined after transfection of Huh7.5 cells. The titers were determined by FFU assays and shown by mean of triplicates ± SEM (*y*-axis). J6/JFH1 was included as a control. *y*-axis break indicates cut-off of the assay. (**B**,**C**): The SNP frequencies (%) were shown for 4d(C5A) viruses. The viruses recovered from indicated passages were NGS analyzed. Only mutations that developed in ≥20% of the virus population are shown. The green boxes indicate the originally introduced mutations in both 4d(C5A)-13m and 4d(C5A)-14m recombinants. The purple box indicates the originally introduced mutation in 4d(C5A)-14m. The region of JFH1-NS5B is indicated with the blue bar.

We generated recombinants with mutations that had frequencies ≥90%, without or with M2002V (M31V, NS5A numbering), since this mutation emerged as putative NS5A RAS during the treatment of genotype 4a ED43 virus with NS5A inhibitors [34], designated 4d(C5A)-13m (Genbank accession number OP555744) and 4d(C5A)-14m (Genbank accession number OP555745), respectively. These recombinant viruses spread efficiently after transfection and produced peak infectivity titers of ~4.7 log_10_ FFU/mL at day 8 (Figure 3A). We performed several passages of 4d(C5A)-13m virus to test whether the mutations were maintained and whether additional mutations emerged. In passage 8, all introduced mutations were maintained. Although the virus acquired additional mutations, they all had frequencies <70% (Figure 3B). The virus produced infectivity titers of ~4.6 log_10_ FFU/mL in the 8th passage. For 4d(C5A)-14m, the recovered virus yielded peak titers of ~4.6 log_10_FFU/mL in the 2nd passage and did not acquire any mutations ≥ 20% while it maintained introduced mutations (Figure 3C).

We next tested whether the 12 mutations in 4d(C5A)-13m, except for JFH1 NS5B mutation Q2851H, also could confer viability of the 4d full-length DH13 recombinant. However, after transfection of Huh7.5 cells with RNA transcripts from pDH13 recombinant with these 12 mutations, we did not observe any HCV antigen-positive cells after 2 weeks of follow-up.

Since there are no genotype 4d infectious systems that have been developed so far, we employed the developed 4d(C5A) recombinant virus to investigate the efficacies of DAAs and NAbs.

### 3.3. Sensitivity of 4d Virus to Direct-Acting Antivirals (DAA)

The recommended DAA regimens for treatment of HCV genotype 4 include NS3/4A protease inhibitors (PIs) grazoprevir and glecaprevir, NS5A inhibitors ledipasvir, elbasvir, velpatasvir and pibrentasvir, and NS5B inhibitor sofosbuvir [9,58]. However, there is limited information about the sensitivity of genotype 4d to these regimens, which has been investigated only in a replicon system [59,60]. Our 4d C5A infectious systems contain authentic DH13 NS3/4A protease and NS5A domains, thus providing useful tools for testing PIs and NS5A inhibitors. However, compared to the predominant aa of other genotype 4 sequences in the Los Alamos database [54], the sequences of our 4d clones contain differences in the NS3 protease domain, but are identical with the predominant sequence of other 4d strains (Table 2).

**Figure 4:**
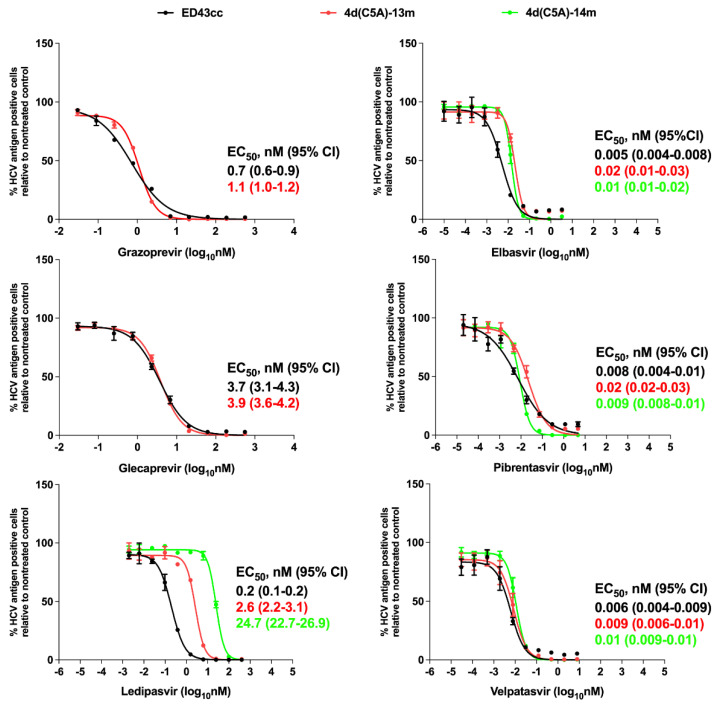
Efficacy of PIs and NS5A inhibitors against HCV genotype 4a (ED43) full-length and 4d(C5A) culture viruses. Huh7.5 cells were seeded on 96-well plates overnight, then infected with the indicated viruses for 24 h. The cells were subsequently treated with specific inhibitors for an additional 48 h before analysis. Values are means of triplicates ± SEM. The ED43cc 4a virus was included for comparison. Mean EC_50_ (nM) values with 95% confidence intervals (95% CI) are shown.

Here, the ED43cc 4a virus was used for comparison [34]. For the PIs, the EC_50_ values obtained for this virus were like those reported previously [34]. In addition, we did not see major differences in sensitivity between the genotype 4a (ED43) and 4d (DH13) viruses. Further, the drug potency against the 4d virus is similar between grazoprevir and glecaprevir (Figure 4).

We have previously shown that NS5A RAS M31V (M2002V) emerged during the treatment of genotype 4a ED43 virus with NS5A inhibitors [34]. The M31 (M2002) is also the predominant aa in HCV genotype 4, including 4a and 4d, but the predominant aa in genotype 4r is L31; the DH13 NS5A domain I was conserved compared to other 4d strains (Table 3). In the present study, we observed the emergence of the mutation M31V (M2002V) during adaptation of the 4d(C5A)-3m recombinant virus (Figure 2). Thus, we investigated whether this substitution could confer viral resistance to NS5A inhibitors. We tested efficacies of NS5A inhibitors ledipasvir, elbasvir, velpatasvir, and pibrentasvir against 4d(C5A)-13m and 4d(C5A)-14m (harboring M2002V) viruses in comparison with genotype 4a ED43cc. The EC_50_ values for ED43cc obtained here are similar with those reported previously [34]. Interestingly, we found that genotype 4d was less sensitive to ledipasvir compared with the 4a virus, with 13-fold increase in EC_50_ values (ED43cc vs. 4d(C5A)-13m) (Figure 4). Importantly, the putative RAS NS5A M31V (M2002V) conferred some level of resistance to ledipasvir with ~10-fold increase in EC_50_ (4d(C5A)-13m vs. 4d(C5A)-14m) (Figure 4). Nevertheless, all tested viruses were inhibited by ledipasvir in dose-dependent manners. Other NS5A inhibitors were all efficient against tested viruses without major differences in EC_50_ values (Figure 4).

### 3.4. Sensitivity of HCV Genotype 4d Virus to Monoclonal and Patient Plasma NAbs

**Figure 5:**
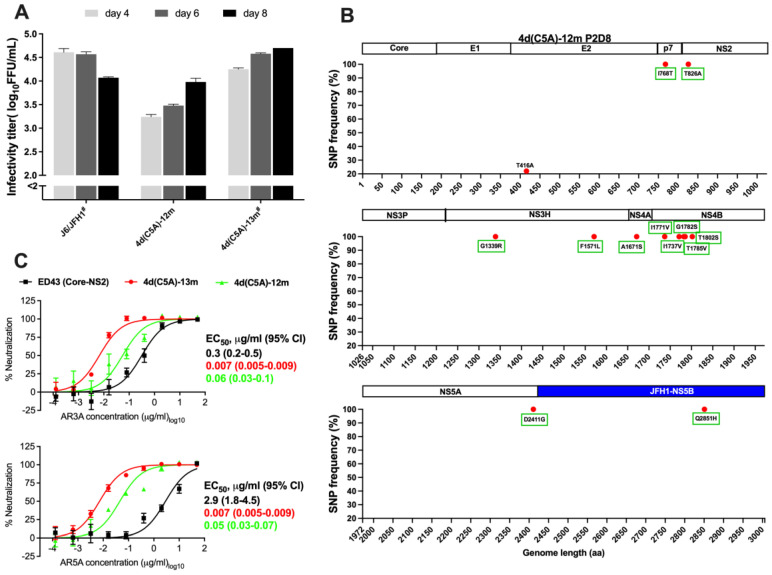
Sensitivity of HCV genotype 4a ED43 Core-NS2 and 4d DH13 Core-NS5A (C5A) recombinant viruses to human monoclonal antibodies, AR3A and AR5A. (**A**): Infectivity titers in supernatants for selected time points of indicated viruses were determined after transfection of Huh7.5 cells (see legend of Figure 3A). ^#^ These data were also shown in Figure 3A. (**B**): The SNP frequencies (%) were shown for 4d(C5A)-12m virus. The virus recovered from the 2nd passage was NGS analyzed. Only mutations that developed in ≥20% of the virus population are shown. The green boxes indicate the originally introduced mutations in 4d(C5A)-12m recombinant. The region of JFH1-NS5B is indicated with the blue bar. (**C**): The indicated viruses were subjected to a 5-fold dilution series of either AR3A (top) or AR5A (bottom) from 50 µg/mL to 0.000128 µg/mL. The virus–antibody mixes and virus only were added to Huh7.5 cells for 4 h prior to washing and addition of fresh medium. At 48 h post-infection, cells were immunostained, and the number of FFUs per well were counted. Values are means of quadruplicates ±SEM normalized to the values of 8 replicates of virus only. The data were analyzed by using four-parameter curve fitting to obtain sigmoidal dose–response curves. Mean EC_50_ (μg/mL) values with 95% confidence intervals (95% CI) are shown.

To our knowledge, no HCVpp or HCVcc systems have been developed for genotype 4d. Thus, our 4d(C5A) infectious system provides the first culture model to test the efficacy of NAbs against this genotype. Among human monoclonal NAbs that have been isolated, AR3A, AR4A, and AR5A target conserved epitopes among HCV genotypes [61,62,63]. Antigenic region (AR) 3 overlaps with the receptor binding domain in E2 and includes residues 427-443 (LNCNDSLNTGFLASLFY) and W529-G530. AR4 and AR5 are conformational epitopes that are dependent on a properly folded E1/E2 heterodimer. AR4 includes the specific residue D698, while AR5 comprises residues R639 and L665 [18,19,20,64,65]. The AR3, AR4, and AR5 are highly conserved across HCV genotypes, including 4a and 4d, and here we showed neutralization susceptibility against these genotype 4 subtypes. Only few variant residues were observed when analyzing these epitope sequences from genotype 4 sequences in the Los Alamos database (Table 4). Here, we chose to analyze the activity of AR3A and AR5A antibodies, which have variation in neutralization potency for different genotypes [61]. The 4d(C5A)-13m recombinant harbors the mutation I414T, and this substitution has been shown to increase viral sensitivity to NAbs [66,67]. Therefore, we tested whether this mutation influences neutralization of genotype 4d. We generated a 4d(C5A)-13m recombinant in which T414 was reverted to I414, designated 4d(C5A)-12m (Genbank accession number OP555743). After transfection, the 4d(C5A)-12m recombinant was viable, but it produced lower infectivity titers compared with those of the original 4d(C5A)-13m virus (Figure 5A), suggesting that I414T influenced fitness of the virus. NGS analysis of the recovered 4d(C5A)-12m virus in the 2nd passage showed that all introduced mutations were maintained, but the virus acquired the additional E2 mutation, T416A, observed at a frequency of 22% (Figure 5B).

In neutralization assays with human monoclonal NAbs AR3A and AR5A, we found that 4d(C5A)-12m was less sensitive to NAbs compared with 4d(C5A)-13m virus with ~8-fold increase in EC_50_ values (Figure 5C). Interestingly, the EC_50_ values for AR3A and AR5A were 40 and 400-fold higher, respectively, for genotype 4a ED43 Core-NS2 virus compared with 4d(C5A)-13m, indicating that genotype 4a ED43 was less NAb sensitive compared with genotype 4d (Figure 5C). Nevertheless, all tested viruses were neutralized by AR3A and AR5A antibodies in a dose-dependent manner.

**Figure 6:**
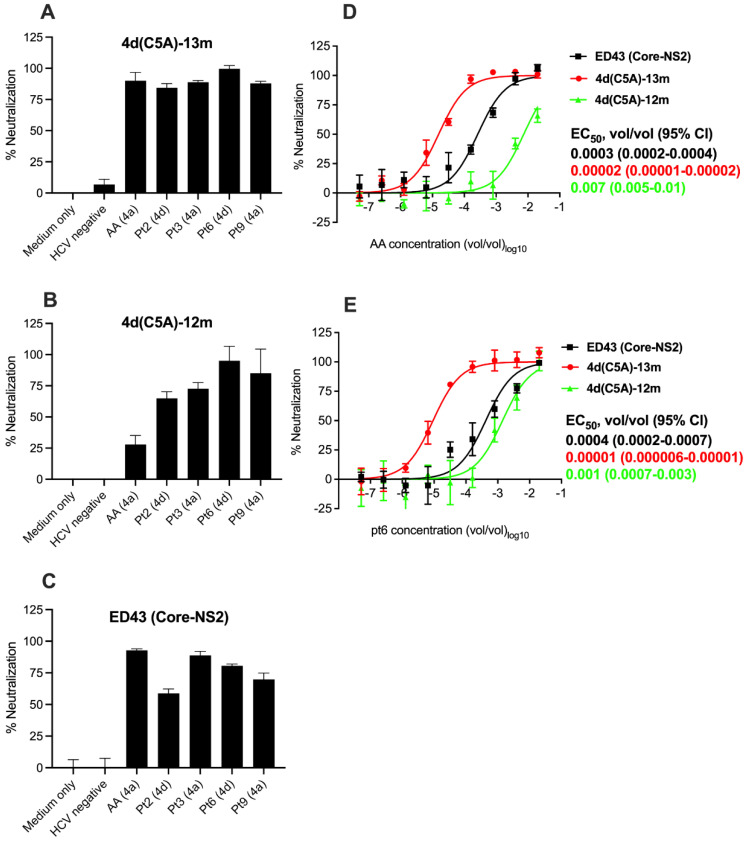
Sensitivity of HCV genotype 4a ED43 Core-NS2 (ED43/JFH1) and 4d DH13 Core-NS5A (C5A) JFH1-based recombinant viruses to plasma obtained from HCV infected patients. The indicated viruses were subjected to a single 1/50 (vol/vol) dilution (**A**–**C**) or a 5-fold dilution series from 1/50 to 1/19,531,250 (vol/vol) of the indicated patient plasma (**D**,**E**). The virus–plasma mixes and the virus only were added to Huh7.5 cells for 4 h prior to washing and addition of fresh medium. At 48 h post-infection, cells were immunostained and the number of FFUs per well were counted. Values are means of quadruplicates ± SEM normalized to the values of 6 replicates of virus only for single-dose neutralization or 8 replicates of virus only for dilution series neutralization. The data of dilution series neutralization were analyzed using four-parameter curve fitting to obtain a sigmoidal dose–response curve. AA, pt3, and pt9: plasma collected from patients infected with HCV genotype 4a. pt2, pt6: plasma collected from patients infected with HCV genotype 4d. Mean EC_50_ (vol/vol) values with 95% confidence intervals (95% CI) are shown.

We next tested neutralization by hepatitis C patient plasma NAbs. We chose to test plasma samples from 3 genotype 4a infected patients, including strain AA [44], and 2 plasma samples from patients infected with genotype 4d. At the dilution 1:50, all plasma samples reduced the number of FFUs by ≥80% for the 4d(C5A)-13m virus (Figure 6A). For 4d(C5A)-12m virus, plasma AA (4a) neutralized by only ~25%, while plasma pt2 (4d) and pt3 (4a) neutralized by ~75% (Figure 6B). The plasma pt6 (4d) and pt9 (4a) had better neutralization capacity, neutralizing this virus by 80–95% (Figure 6B).

For comparison, we also tested this hepatitis C patient plasma panel against ED43 (Core-NS2). At the same dilutions 1:50, plasma pt2 (4d) and pt9 (4a) neutralized this virus by <75%, while other plasma samples could neutralize by ≥80% (Figure 6C). In the control, in which medium or HCV negative plasma were used, <10% neutralizations were observed for the 4a and 4d viruses tested (Figure 6A–C).

To further confirm the neutralization capacity of the plasma, we performed dose -response neutralization assays using plasma AA (4a) and pt6 (4d). Expectedly, both plasma samples could neutralize the 4a and 4d viruses in a dose-dependent manner (Figure 6D,E). Similar to monoclonal NAbs, 4d(C5A)-13m virus was most sensitive to neutralization by plasma (Figure 6D,E). The 4d(C5A)-12m was least sensitive with ~350-and 100-folds increase in EC_50_ values for AA (4a) and pt6 (4d) plasma, respectively (Figure 6D,E). The ED43 (Core-NS2) virus was also less sensitive to plasma neutralization compared with 4d(C5A)-13m virus with ~15-to 40-folds increase in EC_50_ values (Figure 6D,E).

In conclusion, the selected monoclonal and patient plasma NAbs could neutralize the 4a and 4d viruses, but with different capacity, and an adaptive E2 substitution influenced virus neutralization.

### 3.5. Neutralization Sensitivity of Genotype 4d Virus to Serial Plasma Samples from DH13 Infected Patient

**Figure 7:**
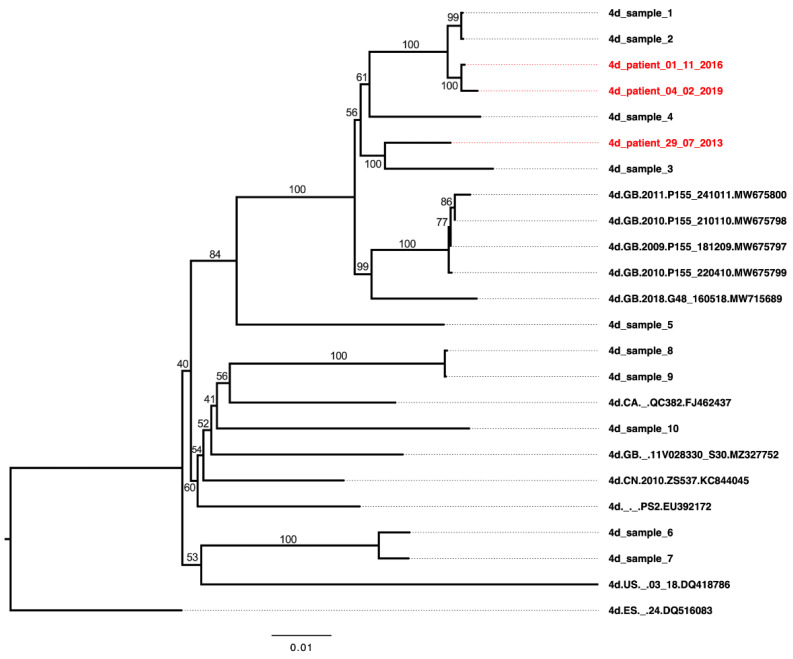
Phylogenetic analysis of near-full length ORF sequences (the first 8937 nts of ORF) of HCV genotype 4d isolates. A total of 11 reference 4d ORF sequences with accession numbers were extracted from the Los Alamos database [54]. These 11 genotype 4d sequences were analyzed also in Table 2, Table 3 and Table 4. All “4d_sample” (numbers 1–10) were near-full ORF sequences from random patients tested in a diagnostic context, and collected and identified, as reported previously [68]. The “4d_patient” samples that are included in this study and shown in Table 5, are marked in red with the sample date indicated; the complete ORF sequence of these samples were determined. The sequences were aligned, and the tree was generated using Jukes–Cantor model and neighbor-joining algorithm. The unit is the number of substitutions per site.

The genotype 4d clone was generated from a patient who was found to be infected with the strain named DH13 in 2013. The patient was treated with DAA grazoprevir/elbasvir in 2015 and had SVR. However, re-infection with another genotype 4d strain was detected in 2016 (Table 5, Figure 7). The patient underwent re-treatment with grazoprevir/elbasvir in 2019. Blood samples was taken again in 2021 and no HCV RNA was detected (Table 5).

These plasma samples provide a valuable source for neutralization analysis of genotype 4d by antibodies that developed during the infection. Thus, we analyzed neutralization capacity against 4d(C5A)-12m virus since this virus does not harbor mutation I414T that could affect neutralization sensitivity of the virus. We also included genotype 4a ED43 Core-NS2 for comparison.

**Figure 8:**
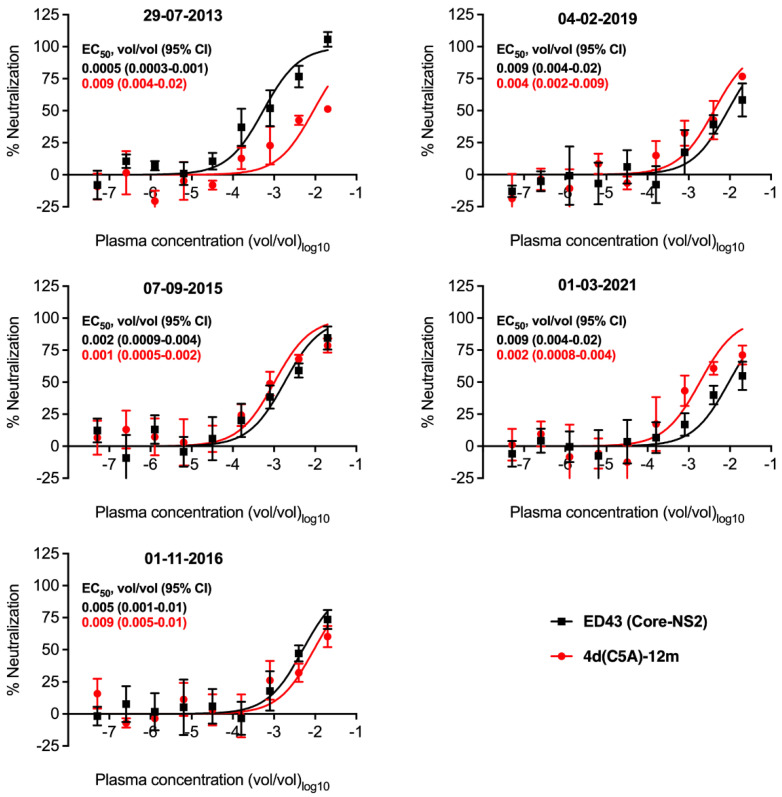
Sensitivity of HCV genotype 4a ED43 Core-NS2 and 4d Core-NS5A (C5A) JFH1-based recombinant viruses to plasma taken at different time points from a patient with chronic HCV genotype 4d infection. The indicated viruses were subjected to 5-fold dilution series from 1/50 to 1/19,531,250 (vol/vol) of plasma collected from the HCV genotype 4d infected patient (initial infection with DH13; reinfected) at the indicated sampling dates. The virus–antibody mixes and virus only were added to Huh7.5 cells for 4 h prior to washing and addition of fresh medium. At 48 h post-infection, cells were immunostained and the number of FFUs per well were counted. Values are means of quadruplicates ±SEM normalized to the values of 8 replicates of virus only. The data were analyzed using four-parameter curve fitting to obtain sigmoidal dose–response curves. Mean EC_50_ (vol/vol) values with 95% confidence intervals (95% CI) are shown.

The plasma collected at 29-07-2013 could neutralize both 4a and 4d viruses (Figure 8). However, the 4d(C5A)-12m virus was less sensitive with ~18-fold increase in EC50_50_ values compared with 4a ED43 Core-NS2 virus (Figure 8). The plasma samples at the next time points, collected 07-09-2015 and 01-11-2016, could also neutralize both viruses with no major differences in virus sensitivity (Figure 8). At the 04-02-2019 timepoint, the neutralization capacity of the plasma sample was maintained at a similar level (Figure 8). At the timepoint 01-03-2021, the plasma could still neutralize both viruses, but the 4d(C5A)-12m virus was slightly more sensitive with ≥4-fold change in EC_50_ compared with 4a ED43 Core-NS2 virus (Figure 8).

In conclusion, the plasma samples collected during the infection of the 4d culture virus source patient maintained activity to neutralize both genotypes 4a and 4d viruses, with relative higher 4d neutralization following reinfection with another 4d strain.

## 4. Discussion

To our knowledge, there is no genotype 4d infectious cell culture system available, and we consequently developed the first system. The in vitro infectious HCV cell culture systems are of great relevance and importance for assessment of NAbs, which could provide insight into vaccine development. Moreover, these systems are useful for testing DAA efficacy and could contribute to optimizing effectiveness of the treatment to overcome drug resistance. Given the increasing prevalence of HCV genotype 4d, the in vitro cell culture system for this genotype is of high relevance and will be important for genotype 4d studies. Based on a JFH1-based genotype 4d Core-NS5A infectious culture system and previously identified adaptive mutations [34] from a genotype 4a ED43 recombinant, the mutations A1671S(NS4A) and T1785V(NS4B) were required for conferring viability of the genotype 4d(C5A) DH13 recombinant [34]. However, addition of mutation D2411G(NS5A) to 4d(C5A) contributed to efficient virus spread in culture. In line with this, it has been shown that mutation A1671S(NS4A) is essential for viral replication, while D2411G(NS5A) is promoting infection [24,69]. Further adaptation of 4d(C5A) virus resulted in emergence of multiple mutations throughout the genome. Among them, mutations T826A(NS2) and F1571L(NS3) (Figure 2) are also observed in genotype 4a ED43 recombinant viruses [34]. The latter suggests that these mutations are universally required for HCV genotypes 4 cultured viruses, which might be of relevance for growing other HCV genotype 4 strains in culture. In addition, in future studies it would be of interest to test whether 4d(C5A)-13m recombinant is viable in vivo.

The reverse engineered patient derived 4d full-length DH13 recombinant was viable in human-liver chimeric mice (in vivo), demonstrating that this consensus sequence is fully viable in a liver organ. However, this recombinant was not viable in cell culture (in vitro), as is the case for most other HCV strains (except for JFH1) [56]. This is an anticipated challenge when culturing HCV and requires cell culture adaptive mutations to facilitate the virus propagation. Aiming at developing a full-length genotype 4d infectious system, we engineered all 4d adaptive mutations in 4d(C5A)-13m (except for a mutation in JFH1-NS5B) into the full-length recombinant. However, we did not observe any HCV antigen-positive cells upon the transfection with RNA transcripts from this clone. Thus, other adaptation strategies must be identified for culturing 4d full-length recombinants in future studies, most likely involving NS5B mutations [24].

Our developed 4d Core-NS5A infectious system contains authentic NS3/NS4A protease and NS5A domain I, which is valuable for evaluating PIs and NS5A inhibitors. However, it does not contain authentic 4d NS5B, thus limiting its use for studying NS5B inhibitors, including sofosbuvir. Among the current PIs used in the clinic, grazoprevir has not been investigated in vitro for genotype 4d, while glecaprevir has been tested using a 4d replicon [60]. In the present study, when comparing with genotype 4a, the efficacy of these PIs against genotype 4d is similar, which support their clinical use for treatment of genotype 4 in general. Grazoprevir showed potency also against other HCV genotypes [31] and has been used in combination with elbasvir for the treatment of genotype 4 infection with SVR rates ≥ 95% [70]. The pan-genotypic glecaprevir also showed activity against HCV genotypes 4a, 4d, and other genotypes, as shown here and in previous studies [60,71]. Except for genotype 4r, the combination of glecaprevir and the NS5A-inhibitor pibrentasvir is recommended for the treatment of HCV genotype 4, including 4a and 4d [9]. The SVR rate is also high (≥93%) for genotype 4 patients [9]. Overall, treatment of genotype 4d infection with these PIs yield high effectiveness both in vitro and in the clinic. For the NS5A inhibitors, the recommended treatment of genotype 4 includes elbasvir (combined with grazoprevir), pibrentasvir (combined with glecaprevir), and velpatasvir or ledipasvir (combined with the NS5B inhibitor sofosbuvir) [9]. In the present study, ledipasvir and elbasvir were tested against genotype 4d for the first time, while velpatasvir and pibrentasvir have also been tested against genotype 4d previously using a replicon system, and high efficacies were reported (~0.002 and 0.003 nM for pibrentasvir and velpatasvir, respectively) [59,60]. Among tested NS5A inhibitors, ledipasvir showed differences in efficacy against genotype 4a compared with 4d. In addition, it also was least potent compared to the other tested NS5A inhibitors. This has important implications for treatment efficacy of genotype 4a and 4d infections in the clinic in which ledipasvir/sofosbuvir has been widely used [72]. Moreover, the putative NS5A RAS M31V (M2002V) that conferred genotype 4d resistance to ledipasvir, had no-to-little effect on efficacy of other tested NS5A inhibitors. In genotype 4a, M31V (M2002V) in combination with L28M (L1999M) and L30H/F (L2001H/F), is associated with virus that escaped from elbasvir or velpatasvir treatments. For ledipasvir, it developed during the treatment, but it was outcompeted by other RASs at the end of treatment [34]. This suggests that as for 4a, the M31V alone is not enough to confer 4d resistance to elbasvir and velpatasvir, and that M31V in combination with other NS5A RASs is needed for conferring viral resistance to those inhibitors.

For antibody neutralization, we found that there were differences in neutralization of genotypes 4a and 4d. For monoclonal NAbs AR3A and AR5A, genotype 4d is more sensitive than the genotype 4a virus. However, for patient plasma, the genotype 4d is overall less sensitive than genotype 4a. In both cases, the tested 4d(C5A)-12m and 4a Core-NS5A viruses did not harbor the E1 and E2 mutations at consensus level, which could alter neutralization sensitivity of the virus [67]. Therefore, these observations suggest that there is a different epitope recognition in the plasma samples compared with monoclonal antibodies AR3A and AR5A. This finding is supported by a previous study in which the differences in neutralization by plasma and monoclonal antibodies were also observed [30]. In future studies, it would be of relevance to test whether the plasma from patients infected with other major genotypes could also neutralize genotype 4d culture virus.

As mentioned, the envelope E1 and E2 mutations could influence neutralization sensitivity of the virus, as investigated previously [67]. In the present study, I414T confers increased neutralization sensitivity by both monoclonal NAbs and patient plasma compared with the virus without this mutation. As reported previously, mutations at this position alter the envelope conformation states resulting in changes in neutralization sensitivity [67]. However, the mutation I414T was acquired during viral adaptation, suggesting that it is needed for increasing infection. Accordingly, we also demonstrated that the addition of I414T increased viral infectivity titers. Supporting this, the 4d(C5A)-12m, which does not contain I414T, is attenuated compared with the 4d(C5A)-13m harboring I414T. Strikingly, 4d(C5A)-12m acquired E2 mutation T416A (~20%) in the 2nd passage culture, which might compensate for the lack of I414T. The link between viral fitness in culture and neutralization sensitivity is not well known, which might be of interest for future investigation.

The 4d DH13 clone was generated from a chronic hepatitis C patient (sample obtained in 2013). However, following treatment induced SVR, this patient was re-infected with another 4d isolate. In our study, we observed clear differences in neutralization when comparing plasma taken in 2013 with plasma collected at later timepoints. The plasma obtained in 2013 had enhanced neutralization of 4a ED43 virus compared with 4d virus, while the neutralization difference was minor for plasma taken by the time of re-infection. It suggests that the heterogeneity of the two 4d isolates that infected the patient influences humoral immune response, resulting in production of different epitope-targeted antibodies, which is highly valuable information for vaccine design. In addition to neutralization differences, the antibody activity seems to be maintained for a long period of time, since we observed no major difference in neutralization for plasma taken more than 7 years apart. In future studies, it would be of interest to test whether the neutralization will be maintained, even though the patient was successfully treated with DAA [73]. These data could help to inform whether NAbs responses could be long-lived following vaccination.

In summary, we have developed an efficient genotype 4d Core-NS5A infectious culture system, providing a highly valuable and relevant model for testing of DAA efficacy and assessment of antibody neutralization. We showed that the recommended PIs and NS5A inhibitors for treatment of genotype 4 infection were efficient against both genotypes 4a and 4d without major differences in efficacy. Both genotypes were also effectively neutralized by monoclonal antibodies and plasma isolated from genotype 4a and 4d patients, though the sensitivity of the virus showed differences. Finally, the neutralization capacity was maintained during and after the infection was cleared by DAA treatment. These data would be useful for the use of DAA in the clinic, as well as for informing vaccine design, which is important for elimination of HCV as a public health threat.

## Figures and Tables

**Table 1:** Primers used to amplify 5′- and 3′-UTR of an HCV genotype 4d strain, as well as primers and probe used for HCV qPCR.

Primer ID	Sequence 5′-3′
4d-1320-R	GCAGTTCTGTTGATGTGCCAGCTC
4d-540-R	CTAGTCGCGCGCACACCCAATCTAG
4drX-9584-RT	CATGATCTGCAGAGAGACC
4dr-8530-F	CTCGACACACTCCAGTCAAC
4drX-9562-R	GTTACGGCACTCTCTGCAGTC
TS-O-00178	GGCCACGCGTCGACTAGTACTTTTTTTTTTTTTTTTTTTTVN
3UTR-9476-F1	GGTGGCTCCATCTTAGCCCTAG
AUAP	GGCCACGCGTCGACTAGTAC
qPCR-forward	AGYGTTGGGTYGCGAAAG
qPCR-reverse	CACTCGCAAGCRCCCT
qPCR-probe	FAM-CCTTGTGGTACTGCCTGA-MGB

V: either dG, dA, or dC; N: dA, dT, dG, or dC at the 3′end. MGB: minor groove binder.

**Table 2:** Differences in amino acids (aa) of the NS3 protease domain (aa 1-188) of analyzed 4d patient sequences and culture infectious clone compared with other HCV genotype 4 sequences.

Predominant aa in GT4 (100)	Predominant aa in GT4a (24)	Predominant aa in GT4d (11)	Predominant aa in GT4r (6)	DH13 Wildtype	4d(C5A)-13m [DH13(C5A)-13m]	4d_Patient 01_11_2016
L13	L	M	L	M	M	M
S15	S	G	G	G	G	G
V18	I	I	V	I	I	I
V33	V	I	V	I	I	I
V48	V	I	I	I	I	I
A101	A	S	A	S	S	S
M147	M	L	M	L	L	L
A150	A	V	A	V	V	V
V151	A	A	V	A	A	A
T185	T	S	T	S	S	S

Note: A total of 100 HCV genotype 4 ORF sequences were extracted from the Los Alamos database (retrieved 28 October 2022) and aligned to determine the predominant aa in genotype 4 compared with our 4d patient sequences and culture infectious DH13 clone. The number of sequences of indicated genotype 4 subtypes are shown in parenthesis. Other genotype 4 sequences include 4f (8), 4k (7), 4b (4), 4p (1), 4g (3), 4m (3), 4q (5), 4c (1), 4o (4), 4n (4), 4t (1), 4l (3), 4v (7), 4s (1), and unassigned subtype 4 (7).

**Table 3:** Differences in amino acids (aa) of NS5A domain I (aa 1-200) of analyzed 4d patient sequences and culture infectious DH13 clones compared with other HCV genotype 4 sequences.

Predominant aa in GT4 (100)	Predominant aa in GT4a (24)	Predominant aa in GT4d (11)	Predominant aa in GT4r (6)	DH13 Wildtype	4d(C5A)-13m [DH13(C5A)-13m]	4d(C5A)-14m [DH13(C5A)-14m]	4d_Patient 01_11_2016
S2	E	R	S	R	R	R	C
V8	V	I	V/I ^#^	I	I	I	I
M31	M	M	L	M	M	V	M
E46	E	V	E	V	V	V	V
I67	I	V	V	V	V	V	V
G98	G	S	G	S	S	S	S
D126	D	E	D	E	E	E	E
L168	L	M	L	M	M	M	M
S174	S	S	S	F	F	F	T
S176	S	T	S	T	T	T	A
S181	S	T	T	T	T	T	T

Note: A total of 100 HCV genotype 4 ORF sequences were extracted from the Los Alamos database (retrieved from 28 October 2022) and aligned to determine the predominant aa in genotype 4 compared with our DH13 clones. See also Table 2 Note. The number of sequences of indicated genotype 4 are shown in parentheses. ^#^ 3 sequences have V, and the other 3 sequences have I.

**Table 4:** Differences in amino acids (aa) of AR3A, AR4A, and AR5A epitopes of DH13 clones compared with other HCV genotype 4 sequences.

Predominant aa in GT4 (100)	Predominant aa in GT4a (24)	Predominant aa in GT4d (11)	Predominant aa in GT4r (6)	DH13 Wildtype	4d(C5A)-12m [DH13(C5A)-12m]	4d(C5A)-13m [DH13(C5A)-13m]	4d_Patient 01_11_2016
I414	I	I	I	I	I	T	I
N434	N	N	Q	N	N	N	N
L438	L	I	I	L	L	L	L
S440	S	S	G	S	S	S	S

Note: A total of 100 HCV genotype 4 ORF sequences were extracted from HCV Los Alamos database (retrieved from 28 October 2022) and aligned to determine the predominant aa in genotypes 4 compared with our DH13 clones. See also Table 2 Note. The number of sequences of indicated genotype 4 are shown in parenthesis.

**Table 5:** Analysis of longitudinal plasma samples from a patient with HCV genotype 4d infections.

Date of Sampling	History of Infection	HCV RNA Titer IU/mL	NGS Analysis	NAb EC_50_ Value (Genotype)
29-07-2013	First infection with genotype 4d detected	5.48 × 10^6^	DH13	0.0005 (4a) vs. 0.009 (4d)
07-09-2015	Before DAA grazoprevir/elbasvir treatment	8.7 × 10^3^	No PCR products ^1^	0.002 (4a) vs. 0.001 (4d)
01-11-2016	New infection with genotype 4d detected	1.9 × 10^6^	New 4d isolate (Genbank accession number OP555739) ^2^	0.005 (4a) vs. 0.009 (4d)
04-02-2019	Before re-treatment with DAA grazoprevir/elbasvir	3.7 × 10^7^	New 4d isolate (Genbank accession number OP555740) ^2^	0.009 (4a) vs. 0.004 (4d)
01-03-2021	Post-treatment	0		0.009 (4a) vs. 0.002 (4d)

^1^ RT-PCR was performed, but we could not detect any products. ^2^ The phylogenetic analysis (Figure 7) shows that these consensus sequences evolve in a separate branch from consensus sequence 29-07-2013 (4d DH13 clone), suggesting this is a re-infection with another 4d strain.

## Data Availability

The data presented in this study are available on request from the corresponding author.
